# Nonpharmacological Therapies for Atrial Fibrillation

**DOI:** 10.4061/2011/718056

**Published:** 2012-04-18

**Authors:** Jane C. Caldwell, Miguel A. Arias, Máximo Rivero-Ayerza, Atul Verma, Adrian Baranchuk

**Affiliations:** ^1^Division of Cardiology (Heart Rhythm Service), Kingston General Hospital, Queen's University, Kingston, ON, Canada K7L 2V7; ^2^Cardiac Arrhythmia and Electrophysiology Unit, Department of Cardiology, Hospital Virgen de la Salud, 45004 Toledo, Spain; ^3^Department of Cardiovascular Medicine, Ziekenhuis Oost-Limburg, 3600 Genk, Belgium; ^4^Southlake Heart Rhythm Program, Division of Cardiology, Southlake Regional Health Centre, Newmarket, Ontario, Canada L3Y 8C3; ^5^Medicine and Physiology, Clinical Electrophysiology and Pacing, Kingston General Hospital, Queen's University, Kingston, ON, Canada K7L 2V7

Whilst atrial fibrillation (AF) is the commonest sustained arrhythmia worldwide, it is also the arrhythmia of the elderly with a lifetime AF risk >20% in the over 80s [1]. We are thus on the brink of an AF pandemic, not only due to our ever-aging population but also as a consequence of our population becoming increasingly obese and diabetic. In the US alone, it is expected that the current AF prevalence of *∼*5 million will at least double by the middle of the century [1]. This represents a huge drain on healthcare resources, not only from the direct management of the arrhythmia but from the management of the associated increased rates of stroke, heart failure, and dementia. 

In the last decade, the care of AF has been revolutionised [2] so that now we can realistically hope to cure some of our AF patients through nonpharmacological therapies such as catheter ablation or surgery. This issue examines the current state of such therapies, points to their strengths, and their weaknesses. One area where improvement is required is the management of AF in the elderly; as eloquently reviewed by L. M. Haegeli and F. Duru, the elderly are often difficult to treat medically because of their multiple comorbidities and are often denied formal anticoagulation from which they stand to gain the most benefit. The elderly more often succumb to the proarrhythmic effects of rhythm management drugs and yet they are poorly represented in all the major trials of catheter ablation.

Catheter ablation is fast becoming the first line care for paroxysmal AF. One exciting new way to administer this therapy is through cryoablation. J. Ruskin et al. reviews how this therapy is similarly as effective as radiofrequency ablation but with less pain and possibly with less acute thrombogenicity. However, it is not as useful in persistent AF, but what does work reliably in these patients is a contentious issue as discussed in the article by K. P. Letsas et al. Here the authors show that persistent AF cannot be treated by PAVI (pulmonary antral vein isolation) alone but what is the plus other remains contentious, especially as so many of the extra manoeuvres increasing the risk of organised atrial tachycardias as per S. Castrejón et al. Such tachycardias are often more troublesome than the original AF, and the financial cost, as well as the emotional trauma, has to be considered when one is weighing up the costs of management by medication in comparison to management by nonpharmacological care as nicely discussed by Y. Khaykin and Y. Shamiss. Another potential cost saving step would be the performance of catheter ablation without interruption of anticoagulation as promoted by P. Santangeli et al.

In the last decade, the incorporation of alternative adjuncts to the traditional “cut and sew” has made the surgical management of AF quicker and less technically demanding. In this issue, we have three excellent reviews on the advancements of surgical management. M. Castella et al. examine the highly successful Cox-Maze III procedure and how it has been developed with new adjuncts, whilst N. Hiari concentrates more on the pathophysiology of AF and how, like catheter ablation, the new adjuncts in surgery are struggling with how best to treat persistent AF. L. Harling et al. discussed each of the adjuncts in turn before remonstrating how one of the major drawbacks in establishing their clinical usefulness is the lack of homogeneity of (i) techniques used and (ii) types of AF treated in the various trials. One area of variation is the ligation of the left atrial appendage to reduce the risk of thromboembolism. The ability to do this procedure as an routine percutaneously procedure is coming ever closer with advances in left atrial appendage occlusion devices as reviewed by T. Contractor and A. Khasnis. 

The paper by K. Mischke et al. reports on their basic science research into rate control of AF by cooling. This phenomenon is attractive in the management of new onset AF in septic patients with low blood pressure as these patients are often resistant to cardioversion and all traditional agents are negatively inotropic. Perhaps in the future, we will be looking at active cooling as already instituted for post arrest patients.

We hope you enjoy this issue that succinctly explores the depth and breadth of currently available nonpharmacological therapies for AF. Such therapies, which offer the potential to cure, will doubtless become more and more essential as we attempt to cope with the Tsunami of AF that is approaching. And so looking to the future, it is going to be both exciting and challenging for interventional electrophysiologists of the catheter and the blade.

*Jane C. Caldwell* *Miguel A. Arias* *Máximo Rivero-Ayerza* *Atul Verma* *Adrian Baranchuk*
